# Clinical Picture and Outcomes in Patients Diagnosed with Brain Abscess

**DOI:** 10.3390/jcm14207237

**Published:** 2025-10-14

**Authors:** Anna Furman-Dłubała, Agnieszka Bednarska, Marek Radkowski, Marcin Paciorek, Joanna Kołodziejska, Tomasz Laskus, Dominik Bursa, Dawid Porowski, Michał Makowiecki, Abdulla Hourani, Martyna Mrozek, Katarzyna Polak, Justyna Kowalska

**Affiliations:** 1 Hospital for Infectious Diseases in Warsaw, Wolska 37, 01-201 Warsaw, Poland; 2 Department of Adults’ Infectious Diseases, Medical University of Warsaw, Żwirki i Wigury 61, 02-091 Warsaw, Poland; 3 Department of Immunopathology of Infectious and Parasitic Diseases, Medical University of Warsaw, Pawińskiego 3C, 02-106 Warsaw, Poland; 4 Doctoral School, Medical University of Warsaw, Żwirki i Wigury 61, 02-091 Warsaw, Poland; 5 Faculty of Medicine, Medical University of Warsaw, Żwirki i Wigury 61, 02-091 Warsaw, Poland; 6 Center for Observational Research in Infectious Diseases, Medical University of Warsaw, Żwirki i Wigury 61, 02-091 Warsaw, Poland

**Keywords:** brain abscess, CNS infection, antibiotic therapy

## Abstract

**Objectives**: The aim of this study was to identify factors that affect the clinical course, outcome, and duration of hospital stay in patients with a brain abscess. **Methods**: Eighty-four inpatients with a confirmed brain abscess were the subjects of this retrospective study. The impact of several factors on the length of hospital stay was evaluated and multiple linear regression analysis was used. **Results**: Several factors affecting the length of hospitalization were identified. These were older age and larger abscess diameter. **Conclusions**: Proper handling of preexisting infections and early consideration of CNS infection, especially in high-risk groups, may reduce the incidence of brain abscess and improve its prognosis. The factors affecting the length of hospitalization shown in this study should be considered upon admission to reduce patients’ unfavorable outcomes.

## 1. Introduction

A brain abscess is a localized intracerebral infection that originates from a focal area of cerebritis, later evolving into a collection of pus circumscribed by a vascular capsule [1]. The infectious causative agents include bacteria, fungi, or parasites; however, in immunocompetent patients, bacteria are responsible for over 95% of cases [2]. Although a brain abscess can arise from the contiguous spread of an adjacent infection, such as paranasal sinusitis, otitis media, and meningitis, it may also originate from distant foci of infection, such as the lungs or heart, or be the effect of penetrating trauma or neurosurgery.

The classic triad of symptoms includes fever, focal neurologic deficits, and a headache, the latter being the most common manifestation of a brain abscess [3]. Patients may present with altered consciousness, seizures, or symptoms of meningitis. Subjects with suspicion of a brain abscess should undergo cranial imaging, preferably diffusion MRI, which is the radiological test of choice, whereas lumbar puncture is not recommended unless symptoms of meningitis are present [4].

Despite diagnostic advancements, brain abscesses continue to pose a clinical challenge, requiring close cooperation between specialists in neurosurgery, neurology, infectious diseases, microbiology, and radiology. The aim of the current study was to analyze factors that affect the clinical course, outcome, and duration of hospital stay in patients with a brain abscess.

## 2. Materials and Methods

Eighty-four patients with a bacterial brain abscess treated at the Department of Adults’ Infectious Diseases at Warsaw Medical University over a period of 12 years (2005–2017) were the subjects of the study. The patients were included in the study based on a review of their medical records and ICD-10 (World Health Organization, Geneva, Switzerland) diagnosis coding by a team of infectious disease specialists. We evaluated the impact of several factors on the length of hospital stay using multiple linear regression analysis. The duration of hospitalization was coincident with the length of intravenous therapy, as after switching to oral antibiotics the patients were discharged. We acknowledge that the length of stay (LOS) measured as a duration of intravenous treatment is influenced by our center’s practices, as the decision to switch to oral antibiotics and to discharge a patient was made after clinical and/or radiological improvement. In our study, we focused on predictors that may hold significant implications for healthcare economics.

The diagnosis of a brain abscess was established on the basis of imaging studies—CT or/and MRI of the brain showing localized parenchymal lesion(s) with peripheral edema and characteristic post-contrast ring enhancement performed in patients presenting with clinical symptoms suggestive of a brain abscess (fever, headache, and/or neurologic deficits), with laboratory findings indicative of infection (elevated inflammatory markers in the blood) or the presence of a primary source of infection, such as otitis media or meningitis. The radiological diagnosis was confirmed either by evidence of a brain abscess observed during a surgical procedure—pus-like material aspirated from the lesion, positive pus culture, or histopathological examination confirming a brain abscess—or by the effects of antibiotic treatment.

Patients with epidural and subdural abscesses (empyema), fungal abscesses, cerebral toxoplasmosis, and CNS tuberculosis were excluded from this study.

The following data were analyzed: demographic data; clinical manifestations, including neurological status on admission; comorbidities; the duration of symptoms prior to admission; laboratory data; probable primary source of infection; number of lesions; etiology; and surgical treatment.

To localize the primary site of infection, the following procedures were carried out: chest X-rays, abdominal ultrasounds, echocardiograms, temporal bone and paranasal sinus CT scans, dental panoramic radiographs, and, if tooth decay was suspected as the source of infection, dentist consultation.

### Statistical Calculations

Statistical analysis of the factors that could have influenced the length of hospitalization was performed in 84 patients. Descriptive analysis was first performed in the form of percentages. We fitted ordinary least squares (OLS) regression with an intercept and heteroscedasticity-consistent (HC3) standard errors. We selected the most relevant variables as predictors that did not have low variance. Correlations were assessed between numeric variables, and CRP was excluded as it was highly correlated with age; nonetheless, no other variables showed high correlations. The observation-to-parameter ratio was 10.5:1 for 7 predictors plus the intercept—consistent with the assumptions of multiple linear regression—in which independence of observation (autocorrelation), normality, and linearity requirements were met before fitting the model. Model diagnostics included the Shapiro–Wilk test for residual normality and Breusch–Pagan test for heteroscedasticity. Two-sided *p* < 0.05 defined statistical significance in the primary analysis.

To evaluate the robustness of our findings, we conducted sensitivity analyses. First, we refitted the primary OLS model after excluding in-hospital deaths. Further, to reduce skew and limit the influence of outliers, we modeled log-transformed length of stay as the outcome using the same covariates.

## 3. Results

### 3.1. Demographic Data and Clinical Manifestations

Descriptive analysis showed that 52 patients (61.9%) were male and 32 patients (38.1%) were female, aged 19 to 83 years, with a mean age of 50.05 (SD 15.50) (Table 1).

The most common symptoms were focal neurologic deficits in 57 patients (67.9%), followed by headache (51 patients, 60.7%) and fever (33 patients, 39.3%). The complete classic clinical triad suggestive of a brain abscess (all symptoms mentioned above) was present in 17 patients (20.2%). The signs of meningitis and seizures appeared in 22 (26.2%) and 20 (23.8%) patients, respectively.

Upon admission, consciousness was assessed using the Glasgow Coma Scale (GCS). Fifty-six patients (66.7%) achieved the maximum score (15 points), in twenty patients (23.8%) the score was 14 points, and in six patients (7.1%) the score was 9–13 points. Two patients (2.4%) were assigned a GCS score of 8, which was the lowest score noted.

### 3.2. Comorbidities

The patients’ comorbidities were evaluated using the Charlson Comorbidity Index. Diabetes was diagnosed in 10 patients (11.9%), 10 patients (11.9%) were receiving immunosuppression due to an autoimmune disorder, chronic alcohol abuse was confirmed in 6 patients (7.1%), and 2 patients (2.4%) underwent splenectomy in the past. The average duration of symptoms prior to diagnosis of a brain abscess was 16 days, ranging from 1 to 360 days.

### 3.3. Laboratory Findings at Baseline

An elevated number of white blood cells (≥10 G/L) was found in 30 patients (35.7%), elevated serum C-reactive protein (≥5 mg/L) was found in 53 patients (63.1%), and thrombocytosis (PLT count ≥ 450 G/L) was found in 5 patients (5.9%).

### 3.4. The Most Likely Primary Source of Infection

Odontogenic infection was the most likely source of infection in 42 patients (50%), followed by paranasal sinusitis in 13 patients (15.5%), and otogenic infection in 9 patients (10.7%). Pneumonia was diagnosed in eight cases (9.5%) and endocarditis was diagnosed in five cases (6%). Histories of neurosurgery and head trauma were present in nine (10.7%) and five patients (6%) respectively. Nineteen patients (22.6%) were concomitantly diagnosed with bacterial meningitis.

### 3.5. Radiological Evaluation

Fifty-three patients (63.1%) had a single brain abscess on radiographic imaging. The average diameter of the abscess was 27.55 mm (ranging from 6 to 72 mm, SD 13.03). A mass effect seen on imaging was noted in 44 patients (52.4%).

### 3.6. Microbiology of Brain Abscesses

Out of 48 patients (57.1%) who were surgically treated, in 41 patients (85.4%) abscess cultures were positive. Twenty-eight different etiological agents were identified, and the most common were *Streptococci* (15.5%), *Staphylococci* (13.2%), and *anaerobes* (10.7%). In nine cases (22%), the etiology of the brain abscess was mixed, as at least two different pathogens were found in aspirate cultures (*Staphylococcus aureus MSSA* and *Enterobacter cloacae*, *Fusobacterium* and *Streptococcus* spp., *Peptostreptococcus* and *Fusobacterium nucleatum*, *Bacteroides* and *Streptococci G*+, *Fusobacterium nucleatum* and *Staphylococcus intermedius*, *Micromonas micros* and *Aerococcus* spp., *Staphylococcus aureus MSSA* and *Enterococcus faecalis*, *Staphylococcus aureus MSSA* and *Peptostreptococcus* sp., and *Enterococcus faecium* and *Enterococcus faecalis*). Among 36 patients treated non-surgically, 5 patients had positive blood cultures and the pathogens discovered were *Staphylococcus aureus MSSA*, *Staphylococcus aureus MRSA*, *Listeria monocytogenes*, and *Enterococcus faecium*.

### 3.7. Treatment

Forty-eight patients (57.1%) underwent surgery (stereotactic aspiration or excision of the brain abscess) followed by antimicrobial therapy, whereas the remaining thirty-six patients (42.9%) received pharmacological treatment only. The average diameter of the abscess lesions among subjects treated by neurosurgery was 31.8 mm (ranging from 13 to 72 mm), whereas in those treated with antibiotics it was 21.4 mm (ranging from 6 to 36 mm). Although retrospectively, clinical criteria were not available, abscess size, location, and the presence of mass effect were the factors for selecting treatment strategy, consistent with standard practices in both our department and the neurosurgical center.

The average duration of intravenous antibiotic therapy was 6.4 weeks (SD 2.58) (ranging from 2 to 13 weeks). The average duration of intravenous antibiotic therapy for patients undergoing surgery was 6.9 weeks, and in non-surgically treated patients it was 5.7 weeks.

The standard intravenous treatment consisted of penicillin G (PEN G), ceftriaxone (CXA), and metronidazole (MTX); however, when staphylococcal infection was suspected, cloxacillin or vancomycin were added in place of penicillin G. If hospital-acquired infection was suspected, broad-spectrum antibiotics (e.g., meropenem and ceftazidime) were added to the treatment regimen. As soon as the result of abscess culture was known, the treatment was specifically modified. Corticosteroid therapy was given to 53 patients (63.1%) with clinical indications, such as mass effect due to significant edema surrounding the abscess.

### 3.8. GOS at the Time of Hospital Discharge

The patients’ outcomes were evaluated while being discharged from the hospital by assigning a score on the five-point Glasgow Outcome Scale (GOS). Sixty-one patients (72.6%) recovered without any neurologic deficits (GOS 5) and fourteen patients (16.7%) had some residual neurologic deficits (GOS 4). Four patients (4.8%) had significant neurologic deficits upon discharge and five patients (6%) died during hospitalization. A GOS score of 1–3 was associated with the average duration of symptoms prior to diagnosis, focal neurologic deficits found on initial physical examination, and multiple brain abscesses, and it was statistically significant with a *p*-value of <0.05 in all of the mentioned factors.

### 3.9. Predictors of the Length of Hospital Stay

Multivariable OLS regression showed that older age and larger abscess size were independently associated with a longer length of stay (LOS). Each additional year of age was linked to a 0.041-week increase in LOS (95% CI 0.007–0.074; *p* = 0.016). Likewise, each 1 mm increase in abscess dimension corresponded to a 0.070-week longer LOS (95% CI 0.021–0.120; *p* = 0.005). Symptom duration, neurologic deficits, multiple abscesses, concurrent bacterial meningitis, and surgical treatment were not significantly associated with LOS in this model. The overall model fit was moderate (R^2^ = 0.485; adjusted R^2^ = 0.426; F = 4.766, *p* = 0.013).

To test the robustness of the primary findings, we re-estimated the models after excluding patients who died in-hospital and using log-transformed LOS. When in-hospital deaths were excluded, both age and abscess dimension remained independently associated with longer LOS: each additional year of age was linked to a 0.037-week increase in LOS (95% CI 0.003–0.071; *p* = 0.034), and each 1 mm increase in abscess size corresponded to a 0.075-week longer stay (95% CI 0.015–0.135; *p* = 0.015). Other covariates were not statistically significant. The model fit was modest (R^2^ = 0.388; adjusted R^2^ = 0.283; F = 2.237, *p* = 0.042).

Using log-transformed LOS yielded consistent results: age (β = 0.006; 95% CI 0.001–0.011; *p* = 0.028) and abscess dimension (β = 0.011; 95% CI 0.003–0.019; *p* = 0.006) remained significant factors. Interpreted on the log scale, these corresponded to ~0.6% longer LOS per year of age and ~1.1% longer LOS per 1 mm increase in abscess size. Neurologic deficits contributed to longer LOS (β = 0.212; *p* = 0.093), while other predictors were not significant. The overall model fit was similar (R^2^ = 0.359; adjusted R^2^ = 0.285; F = 3.234, *p* = 0.005). Together, these sensitivity analyses indicated that the association of older age and larger abscess size with prolonged hospitalization was robust to exclusion of early mortality and accounted for LOS skewness.

**Figure 1:**
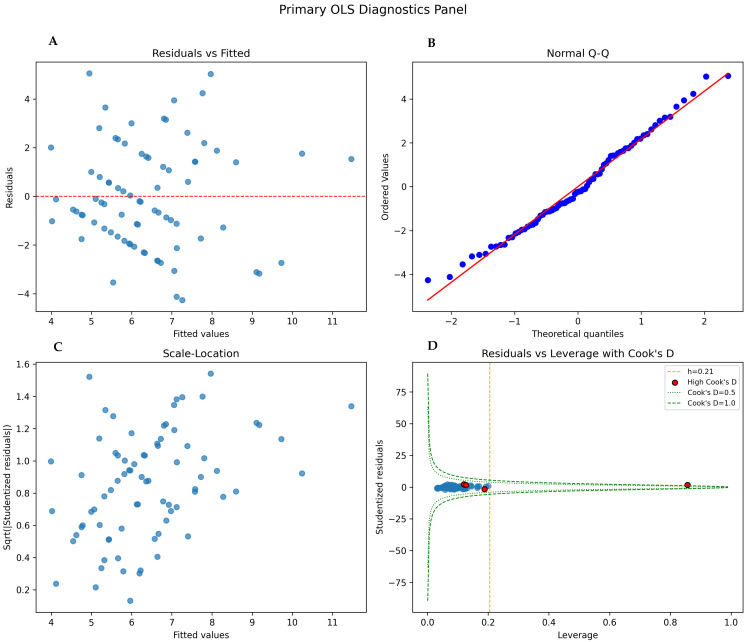
Primary OLS diagnostics for length of intravenous (IV) antibiotic therapy (LOS_weeks) in patients with bacterial brain abscess. Panels: (**A**) Residuals vs. fitted show no systematic pattern; a mild increase in spread at higher fitted values is small and addressed by HC3 robust standard errors. (**B**) Normal Q–Q plot is near-linear, indicating approximately normal residuals (Shapiro–Wilk *p* = 0.455). (**C**) Scale–location plot suggests near-constant variance with only a gentle upward trend, consistent with a non-significant Breusch–Pagan test (*p* = 0.187). (**D**) Residuals vs. leverage identified four observations with Cook’s D > 4/n, all within conventional Cook’s D = 0.5/1.0 contours and with small studentized residuals, indicating limited influence.

Model diagnostics indicated acceptable OLS assumptions: residuals were approximately normal (Shapiro–Wilk *p* = 0.455) with no evidence of heteroscedasticity (Breusch–Pagan *p* = 0.187) and low multicollinearity (all VIFs < 2). A leverage–residual plot flagged four observations with Cook’s D > 4/n, none exceeding conventional influence contours. The model explained 42.6% of LOS variation (adjusted R^2^ = 0.426; F-test *p* = 0.0135). Results were robust in sensitivity, excluding deaths, with log-transformed LOS, and in parametric AFT models (Weibull and log-normal), consistently identifying older age and larger abscess diameter as associated with longer IV therapy (Figure 1, Table 2 and Table 3).

## 4. Discussion

Despite considerable progress in imaging techniques, neurosurgery, and pharmacotherapy over the past decades, the diagnosis and treatment of brain abscesses remain a challenge. The most important and currently unanswered dilemmas revolve around selecting the most effective treatment approach to reduce an unfavorable outcome and the length of hospitalization. The aim of this study was to identify factors that affect the clinical course, outcome, and duration of hospital stay in patients with a brain abscess.

The study population consisted of 84 inpatients with a confirmed brain abscess. Neurologic deficits were the most common manifestations, observed in almost 70% of patients, which was similar to other studies, and were followed in frequency by headache and fever [5]. The high prevalence of neurologic deficits among our patients was likely the consequence of a delayed diagnosis and an advanced stage of the disease. The average diameter of the abscess was almost 3 cm, with mass effect seen in over half of the patients.

Forty-eight of the analyzed patients (57.1%) underwent neurosurgery (stereotactic aspiration or excision of the brain abscess), which was lower than in some other studies in which the proportion of patients undergoing invasive treatment was 70–80% [5,6]. This was partially related to the fact that our study reached back to 2005, when access to more advanced surgical methods was still limited. In particular, the introduction of neuronavigation markedly reduced complications, consequently increasing the number of surgical procedures performed [7]. According to an earlier Polish study covering the years 1996–2005, up to 75% of patients with a brain abscess were disqualified from neurosurgical treatment [8].

The average length of parenteral antibiotic therapy was 6.39 weeks and was consistent with current guidelines [9]. However, the empirical regimens were modified and consisted of penicillin G, ceftriaxone, and metronidazole. Considering the fact that *Streptococci* were the bacteria most commonly cultured from abscesses, and that poor dental conditions were the most common source of infection, broadening the antimicrobial spectrum to penicillin G seemed justified. The oral microflora includes both aerobic and anaerobic *Streptococci* [10]. Additionally, the anaerobic flora was cultured in nine patients (10.7%), and *Fusobacterium* spp. was the most common pathogen (six out of nine patients, 66.7%). These findings were in contrast to another study in which *Bacteroides* spp. (43.4%) was the more common pathogen [11]. This was most likely due to the fact that the source of infection was mostly odontogenic among our patients, whereas in the other study an otogenic source predominated. The anaerobic flora often causes severe tissue damage and/or abscess formation, while diagnosis and treatment are complicated by the slow growth and rising resistance against antimicrobials [12,13]. Consequently, the anaerobic etiology can negatively affect the length of necessary treatment.

Age was an important and independent factor negatively affecting the length of hospitalization, most likely due to immunosenescence and the resulting deficient protection against infection [14]. Older age also increases the risk of misdiagnosing an early stage of CNS infection with neurologic symptoms of other origin, e.g., vascular or drug-induced [15], delaying diagnosis and making treatment more challenging. However, it should be noted that the mean age of our cohort was 50 years, which was less than the age defined by the World Health Organization as older [16]. Brain abscess can occur at any age [17] and, according to our analysis, the length of hospitalization was prolonged by about 0.041-week per year of age. Therefore, the risk of an unexpected length of hospitalization should be considered even in younger patients.

Upon admission, only 17 patients (20.2%) presented with the typical triad of symptoms, namely a headache, fever, and neurologic deficits, in accordance with other studies [3]. Thus, all three symptoms were present in a minority of patients and symptoms were often nonspecific, which, as mentioned in other studies, can cause a delay in diagnosis [17]. The average duration of symptoms was 16 days, which was longer than the 14 days reported by Bodilsen J et al. [18]. Paranasal sinusitis was the second most common source of infection. This kind of infection is known to pose a risk of intracranial complications, and the symptoms of chronic sinusitis can mask the picture of a brain abscess, especially if antibiotics were previously used, resulting in delayed diagnosis [19]. The listed factors could have an impact on recognizing the problem and its diagnosis. The studied individuals were admitted with an advanced stage of infection. It was assumed that this could lead to prolonged treatment and hospitalization (with a 0.070-week longer LOS per every millimetre increase in abscess size).

A poor outcome, defined as death or low GOS score (1–3), was observed in nine patients (10.7%) and was associated with the following factors: average duration of symptoms prior to diagnosis, focal neurologic deficits found on initial physical examination, and multiple brain abscesses. The overall death rate in this study (6%) was similar to or lower than that in other studies [5,10,20,21]. A relatively low mortality rate among our patients could have been due to the high prevalence of Gram-positive bacteria, since individuals with a Gram-negative brain abscess are more likely to experience therapeutic failure [10]. Although findings regarding factors associated with poor outcome were inconsistent in previous studies, focal neurologic deficits upon admission were reported to be a dependent predictor of poor outcome [20,22].

There are several limitations of our study. First, no long-term sequelae of the disease are known. Second, the study was performed in a specialized hospital and therefore patients could be preselected according to certain comorbidities or severity. Third, since this is a retrospective study, the results should be interpreted with caution and require prospective validation in a different cohort of patients. However, our study points out that proper management of preexisting infections, such as sinusitis or periodontitis, and early consideration of CNS infection may play a crucial role in reducing the incidence of brain abscess, improving its prognosis and shortening the length of hospitalization.

## Figures and Tables

**Table 1:** Clinical and demographic overview of 84 patients with brain abscess.

	Number of Patients (%)	Mean (SD)
**Demographics**		
Age (years)		50.05 (15.50)
Sex (male/female)	52/32 (61.9/38.1%)	
**Abscess characteristics**		
Dimensions (mm)		27.55 (13.03)
Multiple abscesses	31 (36.9%)	
*Streptococci*	13 (15.5%)	
*Staphylococci*	11 (13.1%)	
Gram-negative	9 (10.7%)	
Gram-positive	4 (4.8%)	
*Anaerobes*	9 (10.7%)	
*Enterococci*	2 (2.4%)	
**Treatment**		
Total treatment length (weeks)		19.20 (10.97)
Intravenous treatment length (weeks)		6.39 (2.58)
Steroids	53 (63.1%)	
Surgical treatment	48 (57.1%)	

**Table 2:** Multiple linear regression assessing the effect of predictors on length of hospital stay (in weeks) in patients with brain abscess.

Characteristics	Coefficient (95% CI)	*p*-Value
**Age (years)**	**0.041 (0.007–0.074)**	**0.016**
Symptoms duration (days)	0.0127 (−0.039, 0.064)	0.628
Neurodeficits	1.433 (−0.269, 3.136)	0.099
Multiple Abscess	0.934 (−0.215, 2.083)	0.111
**Dimensions (mm)**	**0.070 (0.021, 0.120)**	**0.005**
Bacterial meningitis	−0.219 (−1.315, 0.877)	0.695
Surgical treatment	0.138 (−1.393, 1.668)	0.861

R^2^: 0.485; Adjusted R^2^: 0.426; F-statistic: 4.766 (*p* = 0.013).

**Table 3:** Sensitivity analysis—predictors of outcome: coefficients (95% CI) and *p*-values for models excluding in-hospital deaths and for log-transformed outcome.

Characteristics	In-Hospital Deaths Excluded		Outcome Log-Transformed	
	Coefficient (95% CI)	*p*-Value	Coefficient (95% CI)	*p*-Value
**Age (years)**	**0.037 (0.003, 0.071)**	**0.0342**	**0.006 (0.001, 0.011)**	**0.028**
Symptom duration (days)	0.013 (−0.036, 0.062)	0.5991	0.002 (−0.004, 0.007)	0.601
Neurodeficits	1.372 (−0.398, 3.143)	0.1287	0.212 (−0.036, 0.461)	0.093
Multiple Abscesses	0.765 (−0.508, 2.037)	0.2388	0.143 (−0.035, 0.322)	0.115
**Dimensions (mm)**	**0.075 (0.015, 0.135)**	**0.0149**	**0.011 (0.003, 0.019)**	**0.006**
Bacterial meningitis	−0.287 (−1.710, 1.137)	0.6929	0.016 (−0.218, 0.249)	0.896
Surgical treatment	−0.007 (−1.584, 1.571)	0.9933	0.051 (−0.195, 0.296)	0.684
Model statistics	R^2^: 0.388; Adj R^2^: 0.283; F-statistic: 2.237 (*p* = 0.0419)		R^2^: 0.359; Adj R^2^: 0.285; F-statistic: 3.234 (*p* = 0.005)	

## Data Availability

The data that support the findings of this study are available from the corresponding author upon reasonable request. The data are not publicly available due to institutional policy.

## Abbreviations

The following abbreviations are used in this manuscript:

CNS	Central Nervous System
ICD-10	International Classification of Diseases 10th Revision
CT	Computer Tomography
MRI	Magnetic Resonance Imaging
LOS	Length of stay (hospital)
